# High-resolution dynamic atomic force microscopy in liquids with different feedback architectures

**DOI:** 10.3762/bjnano.4.15

**Published:** 2013-02-27

**Authors:** John Melcher, David Martínez-Martín, Miriam Jaafar, Julio Gómez-Herrero, Arvind Raman

**Affiliations:** 1 Department of Engineering Mathematics, University of Bristol, Bristol BS8 1TR, United Kingdom; 2 ETH Zürich, Department of Biosystems Science and Engineering, CH-4058 Basel, Switzerland; 3 Departamento de Física de la Materia Condensada C-III, Universidad Autónoma de Madrid, 28049 Madrid, Spain; 4 School of Mechanical Engineering and Birck Nanotechnology Center, Purdue University, West Lafayette, IN 47907

**Keywords:** atomic force microscopy, dAFM, high-resolution, liquids

## Abstract

The recent achievement of atomic resolution with dynamic atomic force microscopy (dAFM) [Fukuma et al., *Appl. Phys. Lett.* **2005**, *87*, 034101], where quality factors of the oscillating probe are inherently low, challenges some accepted beliefs concerning sensitivity and resolution in dAFM imaging modes. Through analysis and experiment we study the performance metrics for high-resolution imaging with dAFM in liquid media with amplitude modulation (AM), frequency modulation (FM) and drive-amplitude modulation (DAM) imaging modes. We find that while the quality factors of dAFM probes may deviate by several orders of magnitude between vacuum and liquid media, their sensitivity to tip–sample forces can be remarkable similar. Furthermore, the reduction in noncontact forces and quality factors in liquids diminishes the role of feedback control in achieving high-resolution images. The theoretical findings are supported by atomic-resolution images of mica in water acquired with AM, FM and DAM under similar operating conditions.

## Introduction

Since its inception [1], dynamic atomic force microscopy (dAFM) has proven to be a powerful yet versatile tool capable of operating in media ranging from vacuum to liquids and interrogating samples ranging from stiff inorganic materials to soft biological samples, with nanoscale resolution. Recently, the achievement of atomic-resolution imaging in liquids [2–6] has challenged the accepted belief that high quality factors, which are a hallmark of microcantilever probes in vacuum, are necessary for atomic-resolution imaging [7]. However, atomic-resolution images have now been obtained with several dAFM imaging modes in liquids despite the quality factors being several orders of magnitude smaller than in vacuum.

Several prior works have been dedicated to the understanding of imaging resolution and the role of feedback control in dAFM. Prior efforts to analyze imaging resolution in dAFM have typically focused on the small-amplitude limit in order to establish a relationship between various noise sources in the experimental setup and the minimum detectable gradient of the tip–sample force [1,4,8–9]. However, the optimal imaging amplitude in FM has also been considered [10]. The role of feedback control in dAFM and its stability have been studied largely by using numerical simulations to solve complex systems of nonlinear, integro-differential equations governing the deflection of the oscillating probe subject to feedback control [11–13]. Kilpatrick et al. [14] neglected tip–sample forces in order to provide an estimate for stable control parameters in FM. To improve imaging resolution in liquids, Q-controlled dAFM, which uses feedback control to manipulate the effective quality factor of the oscillating probe, has been proposed [15–16]. However, the merits of this approach for improving imaging resolution are still under question [17].

In this article we present a combined theoretical and experimental study of high-resolution imaging in liquid media with various dAFM imaging modes. The method of periodic averaging [18] is used to simplify the fast-time-scale equations governing the deflection of the oscillating probe by slow-time-scale, averaged equations that govern the amplitude and phase lag of the oscillation. The averaged equations provide a natural starting point for the analysis of closed-loop dAFM imaging modes, which are ultimately designed to regulate the amplitude and phase lag of the oscillating probe rather than its time-varying deflection. From the approximate theory, we explore performance metrics for dAFM imaging modes, such as (i) force sensitivity and resolution, (ii) detection bandwidth, (iii) disturbance mitigation and (iv) imaging stability. In support of our findings, we demonstrate atomic-resolution images of mica in water with FM, AM and DAM under similar operating conditions.

## Analysis of closed-loop dAFM imaging modes

Conventional dAFM imaging modes use a microcantilever probe with a sharp tip affixed to the free-end, which is made to oscillate near its fundamental resonance in close proximity to a sample. Through the influence of tip–sample forces, the presence of the sample is detected in the oscillations of the probe. Let *z* denote the nominal separation between the tip and sample in the absence of tip–sample forces. The realization of a dAFM imaging mode follows from the implementation of a separation regulator that uses *z* as a controlled input for a feedback regulator designed to maintain the amplitude and/or phase lag of the oscillation. The actuation of *z* is implemented by a piezo actuator. The values of *z* that satisfy the regulation objective are interpreted as the topography of the sample. The simplest dAFM imaging mode is AM, where the amplitude is maintained by the separation regulator, while the phase lag of the oscillation is free to vary. Imaging modes with more complex feedback architectures, such as FM and DAM, will be described later in this section.

In order to establish the performance metrics for high-resolution imaging, we start with the equation of motion describing the time-varying deflection *x*(*t*) of the probe tip in the presence of tip–sample forces given by

[1]
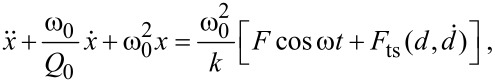


where ω_0_, *Q*_0_ and *k* are the unperturbed natural frequency, quality factor and stiffness of the probe, respectively, and *F* is the excitation force [19]. *F*_ts_ is the tip–sample interaction force, which depends explicitly on the tip–sample gap *d*(*t*) = *z* + *x*(*t*) and its rate 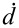. In the absence of tip–sample forces with ω = ω_0_ and *F* = *F*_0_, the tip oscillates with an unconstrained amplitude *a*_0_ = *F*_0_*Q*_0_/*k*. *F* = *F*_0_ and ω = ω_0_ are fixed in AM, but *F* and ω are adjusted by feedback regulators in FM and DAM.

The solution of Equation 1 can separated into two parts, the first being the equilibrium deflection x* in the absence of the excitation force, and, the second being oscillation about x*. At each *z*, x* is found by setting 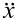 = 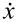 = 0 in Equation 1 and satisfies

[2]
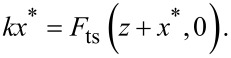


The tip–sample forces are often characterized by an attractive (*∂F*_ts_/*∂d* > 0), noncontact regime when *d* is sufficiently large, which gives way to a repulsive (*∂F*_ts_/*∂d* < 0), contact regime as *d* is reduced. If *k* < *∂F*_ts_/*∂d*, then for some *z*, x* will be bistable. This results in one stable equilibrium for both the noncontact and contact regimes. In this case, a spontaneous transition from the noncontact equilibrium to the contact equilibrium, or *snap-in*, can occur [20]. The snap-in instability is avoided if the equilibrium deflection is monostable, which occurs when either *z* is sufficiently large for a given *k* or when *k* exceeds the maximum gradient of *F*_ts_ [10,21].

The model for the probe dynamics in Equation 1 can be simplified through the use of the method of first-order averaging [22–23]. To this end, consider the overall motion with excitation to be represented by *x*(*t*) = x* + *a*(*t*)cos[ω*t* − 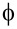
(*t*)], where *a*(*t*) and 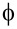
(*t*) are the time-varying amplitude and phase lag, respectively [24]. An autonomous equation describing the dynamics of *a* and 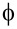 becomes:

[3]
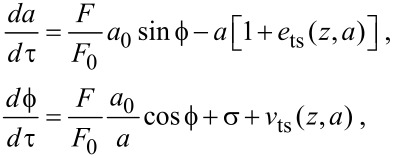


where *τ* = ω_0_*t*/2*Q* and σ = *Q*_0_[(ω/ω_0_)^2^ − 1] is the frequency shift scaled by the half-power bandwidth of the resonance. The nonlinear tip–sample forces are captured in Equation 3 by the functionals

[4]
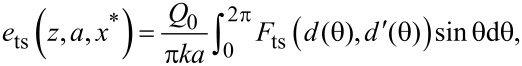


[5]
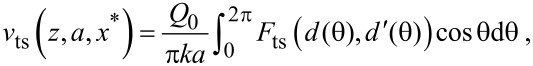


where *d*(θ) = *z* + x* + *a*cosθ and *d*^′^(θ) = −*a*sinθ. *e*_ts_ is the energy dissipated during the tip–sample interaction and *v*_ts_ is the virial of the tip–sample interaction [25]. The virial is related to the kinetic energy stored in the oscillating probe through the virial theorem [26] and is a measure of the maximum potential energy stored in the tip–sample interaction during an oscillation. Moreover, by introducing a specific model for *F*_ts_, a relationship between *v*_ts_ and the interaction potential can be established [27]. Finally, both *e*_ts_ and *v*_ts_ have been nondimensionalized by the energy dissipated by the media during an oscillation cycle *E*_med_ = π*ka*^2^/*Q*_0_. In one form or another, these parameters are ubiquitous in perturbation analysis of dAFM [22–23,26–28].

Equation 3 captures the transient response *a*(*τ*) and 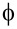
(*τ*) of the oscillating probe. In addition to providing an approximate relationship between the experimental observables and the tip–sample forces, this feature accommodates the study of stability and detection bandwidth. The transient response of both the amplitude and phase lag have a nominal characteristic time scale of 2*Q*_0_/ω_0_ in the absence of tip–sample forces or feedback control. The equilibrium solutions a* and 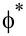 of Equation 3 approximate steady-state, harmonic-oscillation solutions to Equation 1 with constant amplitude and phase lag, oscillating about the equilibrium deflection x*. Note that we have included the dependence on x* in Equation 4 and Equation 5 for completeness. However, unless the equilibrium deflection is bistable, i.e., near snap-in, x* can be neglected in the analysis. This assumption is carried forward, and, in the subsequent analysis, we write *v*_ts_(*z*,*a*) and *e*_ts_(*z*,*a*).

Setting 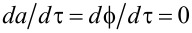 in Equation 3, we arrive at the equations governing the steady-state amplitude and phase lag

[6]
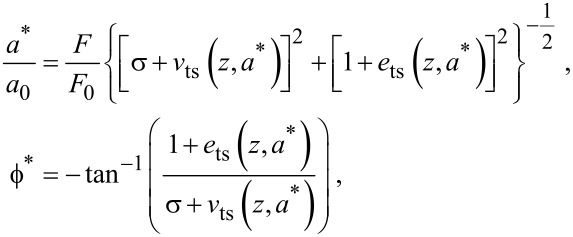


where the asterisk denotes the steady-state. The stability of the steady-state oscillation is determined by Equation 3.

The nonlinear terms *v*_ts_ and *e*_ts_ introduce the possibility of coexisting stable solutions to Equation 6, even when the equilibrium deflection is monostable [21,29–30]. When the equilibrium is bistable, three coexisting stable oscillation states are possible [31]. Such nonlinear phenomena are of considerable practical importance in dAFM and have been studied extensively in the literature [32–34].

Next, we introduce the feedback architectures that define the AM, FM and DAM imaging modes. AM is modeled with Equation 3 by setting *F* = *F*_0_, σ = 0, and introducing a separation regulator that manipulates *z*:

[7]
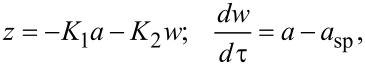


where *K*_1_ and *K*_2_ are gain parameters. The control effort in Equation 7 consists of a proportional controller *K*_1_*a* and an integral regulator *K*_2_*w*, which ensures a* = *a*_sp_. By substituting *F* = *F*_0_, σ = 0 and a* = *a*_sp_ into Equation 6, it can be shown that the AM topography reflects a combination of the *e*_ts_ and *v*_ts_ and the resulting phase lag reveals the relative magnitude of the two. However, it is important that the issue of co-existing oscillation states persists in AM allowing the controller to spontaneously switch between stable states [30].

The FM and DAM imaging modes have more complex feedback architectures than AM. The original implementation of FM conceived by Albrecht et al. [8] used a self-excitation scheme where the excitation signal was generated by applying a phase shift to the deflection signal and the oscillation amplitude was maintained by a regulator. Alternatively, an externally generated, excitation signal and lock-in amplifier can be implemented to maintain a resonant excitation with constant oscillation amplitude [14]. The latter will be the focus of the present analysis. Both FM and DAM incorporate these auxiliary regulators that operate independently from the separation regulator:

[8]
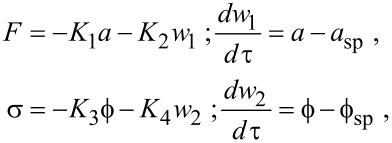


where *K*_1_ − *K*_4_ are gain parameters, *a*_sp_ = *a*_0_ and 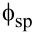 = π/2. The auxiliary regulators ensure that at steady-state, *e*_ts_ is captured in *F* and *v*_ts_ is captured in σ. While both the self-excited and externally excited implementations achieve the same objective, there are some differences in the detection bandwidth and measurement noise. These issues will be discussed briefly in the following section.

The auxiliary feedback regulators essentially have complete control over the oscillations of the probe through their independent manipulation of amplitude and phase lag. When equilibrium deflection x* is monostable, coexisting of stable oscillation states, a* and 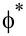 are eliminated by the integral regulators, while the stability and transient settling time can be controlled completely by the proportional controllers. It is straightforward to prove these results by substituting Equation 8 into Equation 3 and solving for the equilibrium points and the eigenvalues of the Jacobian matrix or by using standard tools for control theory [18]. Limitations in the control of the amplitude and phase lag are introduced only after incorporating the finite bandwidth of the amplitude and phase lag measurements into the model [14]. However, we note that instabilities persist when the equilibrium deflection x* is bistable since the auxiliary regulators control the amplitude and phase lag but have no control over x*.

The separation regulator in FM actuates *z* in order to maintain the frequency shift σ according to

[9]
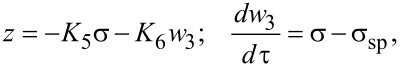


where *K*_5_ and *K*_6_ are gain constants, and σ_sp_ is the set-point frequency shift. At equilibrium in FM, the topography is purely a reflection of the virial of the interaction and the dissipation is measured in the corresponding excitation force signal.

The separation regulator in DAM actuates *z* in order to maintain the excitation force according to

[10]
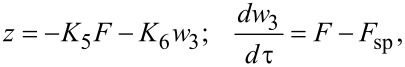


where *K*_5_ and *K*_6_ are gain parameters and *F*_sp_ is the force at the set-point. At equilibrium in DAM, the topography is purely a reflection of the dissipation, and the virial is captured by the corresponding frequency shift. In this respect, DAM can be regarded as the complementary mode to FM.

**Figure 1:**
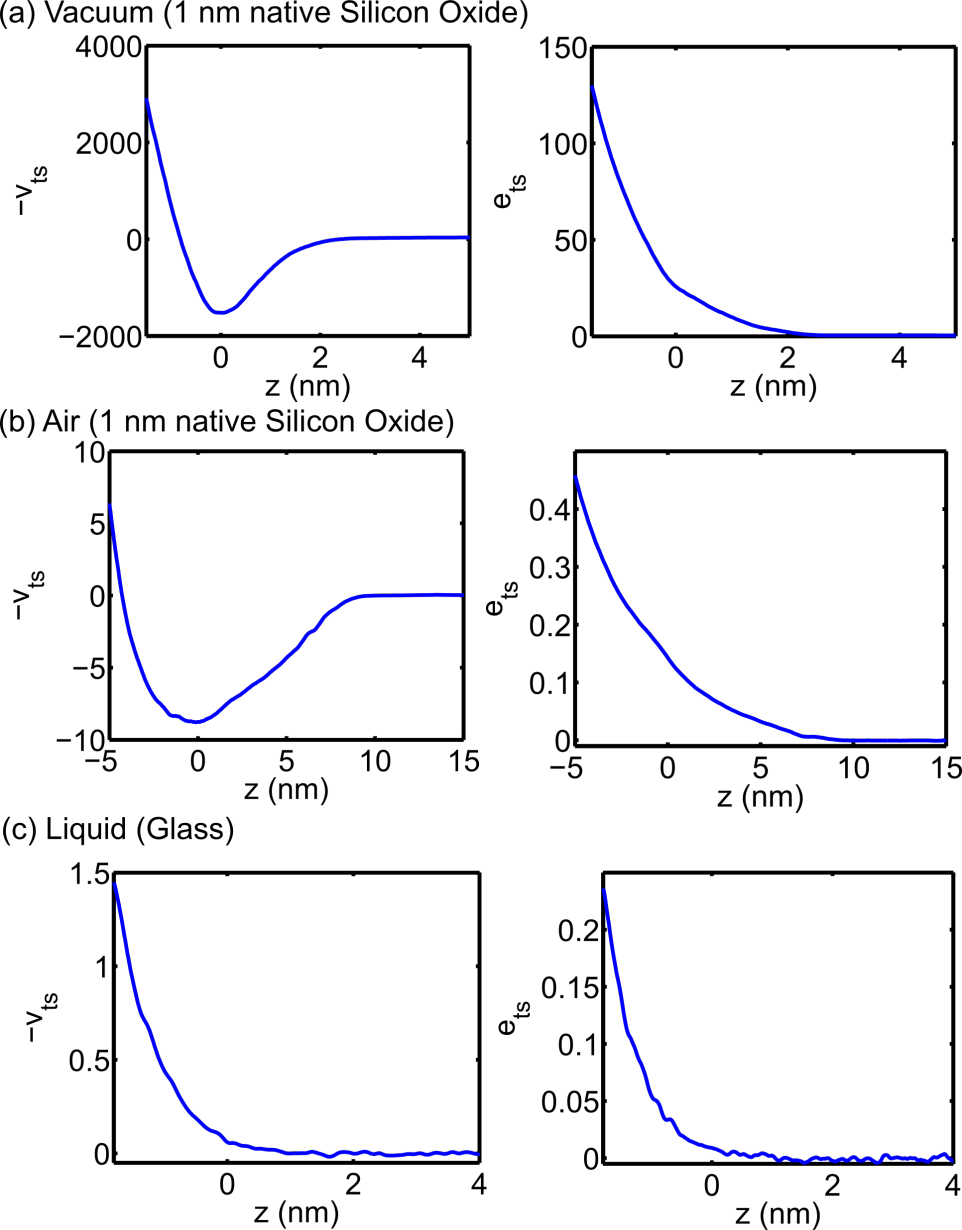
Experimental tip–sample virial *v*_ts_(*z*,*a*_sp_) and dissipation *e*_ts_(*z*,*a*_sp_). Data acquired in vacuum on a silicon sample with 1 nm native silicon oxide (a) in vacuum and (b) in air. (c) Data acquired in deionized water on a glass substrate. The set-point amplitudes were 8.5 nm, 8.6 nm, and 1.5 nm, respectively. See the Methods section for additional information.

At this juncture, it is instructive to introduce some experimental data highlighting some of the key differences between dAFM operation in vacuum, air and liquid. In Figure 1, *e*_ts_ and *v*_ts_ are measured under typical operating conditions in vacuum, air and liquid with an oscillating probe that is controlled by the auxiliary feedback regulators in Equation 8 while *z* is displaced by a piezo actuator (see Methods for additional information). The coordinate *z* is shifted such that *z* = 0 is located approximately at the boundary between the contact and noncontact regimes. For high-resolution imaging, *z* is maintained in the neighborhood of *z* = 0. In vacuum, large long-range noncontact tip–sample forces result in *e*_ts_ >> 1 and |*v*_ts_| >> 1 at imaging distances from the sample. Consequently, the oscillations of the probe are strongly influenced by the presence of tip–sample forces in vacuum. On the other hand, *e*_ts_ and *v*_ts_ are on the order of unity in air and small compared to unity in liquid.

Before proceeding, we will briefly address the issue of higher harmonics in liquids. Early work on dAFM in liquids showed that significant higher harmonic distortions in the oscillation waveform could provide additional channels for compositional mapping [35]. More recently, it was discovered that the use of soft microcantilevers (≤1 N/m) with quality factors close to unity resulted in higher harmonics from higher eigenmodes [36–37]. The present theory does not extend to soft microcantilevers in liquids. However, from prior work, we can expect that the primary difference for soft microcantilevers is that the dissipation reflects the energy lost to higher harmonics [38–39].

## Performance metrics for high-resolution imaging in dAFM

Using the mathematical framework developed in the previous section based on the method of first-order averaging, we now address the question of high-resolution imaging in liquid, despite the low quality factors. We note that while the chemical makeup and atomic configuration of the tip and sample are important considerations for high-resolution imaging, the focus of this article is on the dAFM instrumentation. Specifically, we investigate the performance metrics for high-resolution imaging in dAFM, including (i) force sensitivity and resolution, (ii) detection bandwidth, (iii) disturbance mitigation and (iv) stability in dAFM modes.

### Force sensitivity and resolution

To understand how atomic-resolution imaging is possible in liquids despite the low quality factors, we first examine the sensitivity of the oscillating probe to tip–sample forces. For high-resolution imaging in all dAFM imaging modes, the effect of small tip–sample forces between the foremost atom of the tip and the substrate must be detected in the steady-state amplitude and/or phase lag. Therefore, we are interested in the sensitivity of the steady-state amplitude and phase lag to a small perturbation to the total tip–sample force.

The tip–sample forces are limited in magnitude for a given length scale. These considerations are captured elegantly by an exponential function given by [10]

[11]
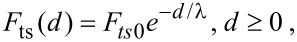


where λ is the characteristic length scale and *F_ts_*_0_ is the magnitude corresponding to *d* = 0. The magnitude of *F*_ts_ is limited by requiring *d* ≥ 0. Following [10], an approximate expression for the *v*_ts_, which holds for an arbitrary amplitude, is given by

[12]
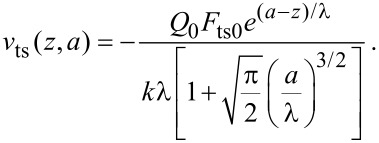


Choosing λ appropriately allows Equation 11 and Equation 12 to approximate a variety of tip–sample forces. Forces at the atomic scale are captured by λ ≈ 1 Å.

The simple model for the tip–sample force in Equation 11 is conservative. Dissipative components of the interaction are more complex in nature and less understood. At the atomic scale, energy may be dissipated from the bulk motion of the tip due to spontaneous transitions between multistable configurations of the nearest atoms of the tip and sample [40–41]. To incorporate dissipation in a simplistic manner, we allow *e*_ts_ = −μ*v*_ts_, where μ is a proportionality constant. From the data in Figure 1, this appears to be a reasonable approximation when the force is unidirectional, which is the case for Equation 11.

Next, we perturb the steady-state amplitude and phase lag about their equilibrium by introducing a small variation in the tip–sample force. This is achieved by perturbing *F_ts_*_0_ by a small amount δ*F*_ts_. Using a first-order Taylor approximation of Equation 6 establishes a relationship between the amplitude and phase lag resolution, δ*a* and 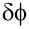 = *δa*/*a*, respectively, and the force resolution, given by

[13]
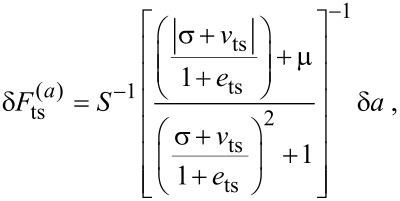


[14]
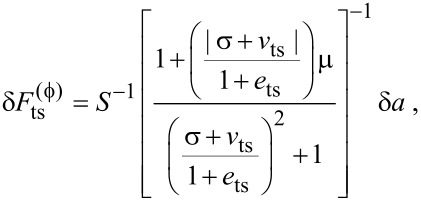


where

[15]
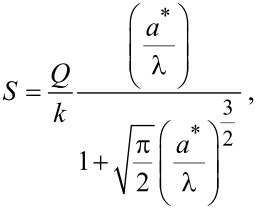


and 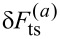 and 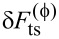 are the force resolutions in the amplitude and phase-lag measurements, respectively, and *Q* = *Q*_0_/(1 + *e*_ts_) is the effective quality factor.

Equation 13 and Equation 14 can be combined into a single approximation for force resolution in dAFM with the following approximation. Note that conventional dAFM modes are designed to excite the probe near its effective resonance frequency in the presence of the tip–sample forces. Thus, we argue that the term (σ + *v*_ts_)/(1 + *e*_ts_) should be on the order of unity or smaller. It follows that the bracketed terms in Equation 13 and Equation 14 are on the order of unity allowing *S* to approximate the sensitivity of both the amplitude and phase-lag measurements to tip–sample forces. The force resolution can be approximated simply by δ*F*_ts_ = *S*^−1^*δa*.

**Figure 2:**
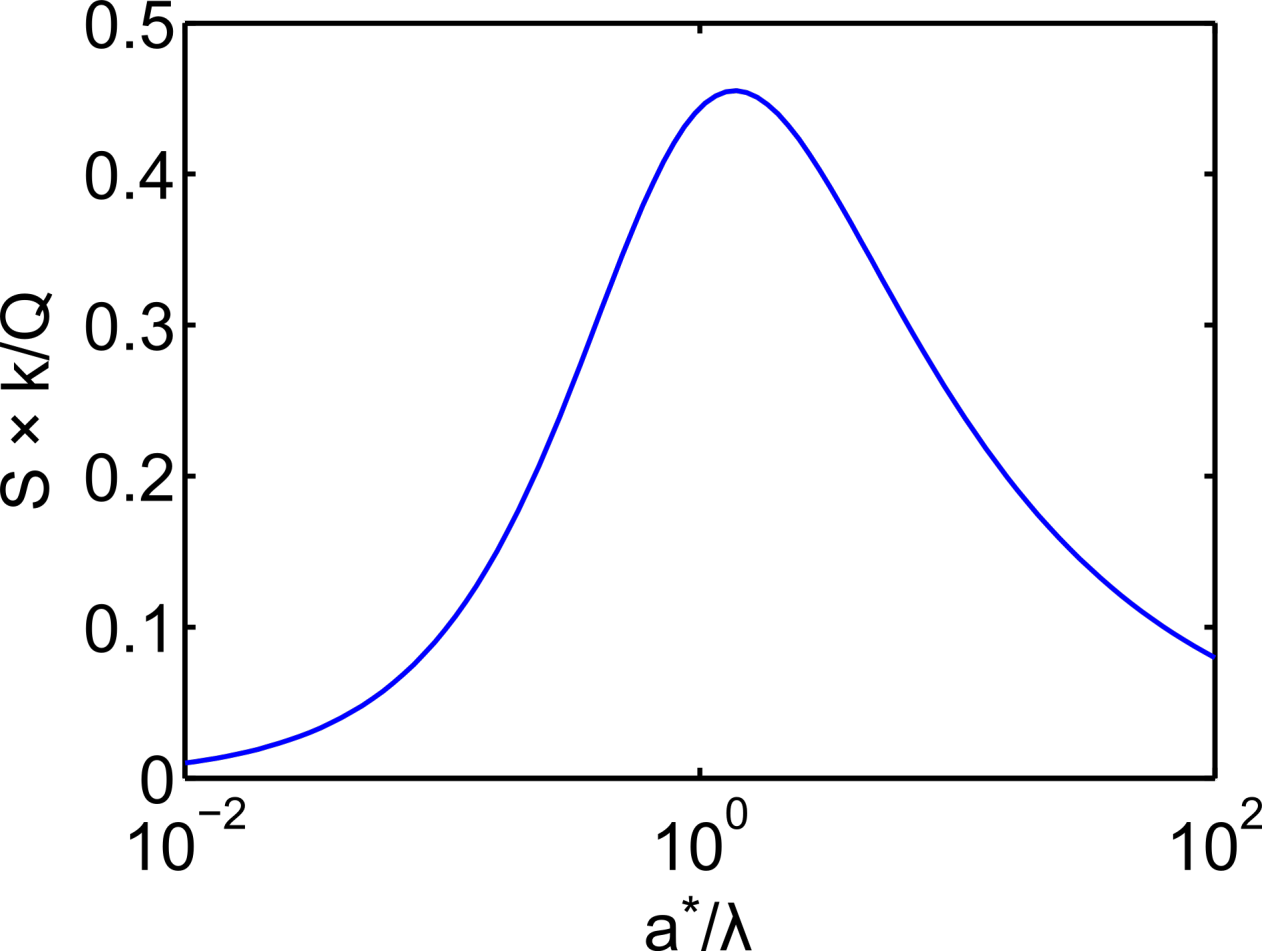
Plot of the normalized force sensitivity *S* × *k*/*Q* versus normalized amplitude a*/λ, where λ is the characteristic length scale of the interaction. The maximum of *S* occurs at a* ≈ λ*.*

Figure 2 plots the normalized force sensitivity *S* × *k*/*Q* versus the normalized amplitude a*/λ. We note that much of the prior work of sensitivity and resolution in dAFM has linearized the tip–sample force to determine the minimum detectable force gradient [8]. Such analyses predict that force resolution in dAFM improves as the amplitude is increased. However, Equation 15, which holds for an arbitrary amplitude, predicts a global maximum in the force sensitivity for a* ≈ λ (see Figure 2). This result is consistent with the analysis of FM by Giessibl et al. [10] but applies to all conventional dAFM modes.

To estimate the force resolution we must also obtain some estimate of the resolution of the amplitude measurement δ*a*. Two important noise sources that contribute to δ*a* are the thermal noise and deflection-sensor noise. In light of recent efforts to reduce the deflection-sensor noise [42], we will focus on the thermodynamic lower limit of δ*a*, which can be approximated by [43]

[16]
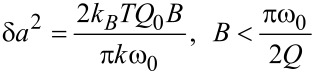


where *B* is the measurement bandwidth, *k*_B_ is the Boltzmann constant and *T* is the temperature. Setting *B* = πω_0_/2*Q*_0_ extends the approximation in Equation 16 to large measurement bandwidths. However, the bandwidth restriction does not apply to self-excited FM [8,44]. Allowing *B* >> ω_0_/*Q* suggests that self-excited FM has the potential to be noisier than externally excited FM; however, this is a topic of ongoing debate [45–46].

Using Equation 16 to approximate δ*a* yields to the following expression for δ*F*_ts_:

[17]
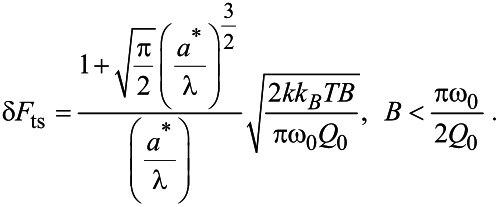


In the case of small amplitudes a* << *λ*, substitution of


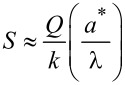


and δ*F*_ts_ ≈ λδ*k*_ts_ Equation 17, where *k*_ts_ is the tip–sample gradient, yields

[18]
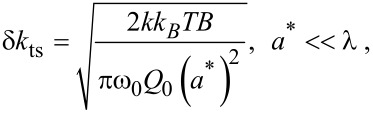


which is essentially the result obtained by Albretch et al. [8]. However, of the two expressions, only Equation 17 captures the range of amplitudes applicable to high-resolution imaging.

The present analysis of the force sensitivity in dAFM begins to shed light on how high-resolution imaging is possible with dAFM in liquids despite the low quality factors. Table 1 lists the force sensitivity *S* and resolution δ*F*_ts_ for several prior works demonstrating atomic resolution in vacuum and liquid environments. While *Q*_0_ degrades by four orders of magnitude in liquid, the force resolution, in some cases, is of the same order of magnitude. Moreover, the force resolution approximated from Giessibl et al. [47] is in accordance with all the measurements in liquids. This result is the direct consequence of the small attractive forces in liquids on clean, hard surfaces, such as mica, that allow *a*_sp_ ≈ λ with a probe that is much softer than the tuning fork [2]. In vacuum, strong attractive forces cause the tip to snap into the surface when oscillation amplitudes are small.

From the data in Table 1, we must also entertain the possibility that force resolution is not necessarily the limiting factor for imaging resolution. The highest resolution images are achieved with the qPlus sensor in [47], which has the lowest force resolution amongst the references in vacuum. If the force sensitivity meets some minimal requirements, the imaging resolution may be limited by other factors, such as the imaging stability [48]. Furthermore, the minimal requirement for force resolution may be less in liquids compared to vacuum where stable images can be acquired in the contact regime.

**Table 1:** Parameters in atomic-resolution imaging in vacuum and liquid medium. *S* (Equation 15) and δ*F*_ts_ = *S*^−1^δ*a* (Equation 17) where calculated assuming λ = 1 Å, *T* = 300 K and *B* = 1 kHz, and *Q* = *Q*_0_ and a* = *a*_sp_.

Mode	Med.	*k* (N/m)	a* (nm)	*Q*_0_	*B*_osc_ (Hz)	log_10_*S*	log_10_*δF*_ts_	Ref.

FM	Vac.	17	34	28,000	2	1.9	−11.4	[49]
FM	Vac.	41	14.8	38,000	2	1.8	−11.6	[50]
AM	Vac.	60	0.2	550	15	0.61	−11.5	[51]
AM	Vac.	1600	0.28	18,000	50	0.58	−12.2	[52]
FM	Vac.	1800	0.8	4000^a^	15	−0.22	−10.8	[47]
FM	Liq.	37	0.33	23	3000	−0.62	−10.7	[2]
PM^b^	Liq.	19	0.59	5.8	12,000	−1.0	−10.5	[3]
AM	Liq.	0.76	0.5	2^c^	1300	−0.057	−10.3	[5]
FM	Liq.	30	0.59	8	8100	−1.1	−10.4	[4]
FM	Liq.	26	0.11	8.3	8400	−0.84	−10.6	[6]

^a^taken from [48]*.*^ b^phase modulation. ^c^taken from our own data.

### Detection bandwidth

The overall detection bandwidth in dAFM can be limited by the bandwidth of the amplitude and phase measurements, or the transient response, or the response of the oscillating probe. Drift in dAFM, for example arising from the piezo actuators controlling the image raster, imposes a minimum scan speed and corresponding detection bandwidth requirement for high-resolution imaging. Giessibl et al. [10] approximate the required detection bandwidth as 1 kHz. From our own experiments (see Methods section), we also estimate that the required bandwidth is on the order of 1 kHz for high-resolution imaging; however, we can expect variability depending on the experimental setup.

Detection of tip–sample forces in dAFM requires that the oscillating probe reaches a steady-state and the separation regulator achieves its objective. Thus, the detection bandwidth is limited by the transient settling time of the oscillating probe. As we have discussed, the amplitude and phase lag evolve on a characteristic time scale of 2*Q*_0_/ω_0_, which corresponds to the ring-down time of the probe in the absence of tip–sample forces and without feedback control. The corresponding bandwidth (rad/s) is

[19]
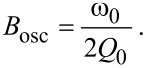


However, it is important to note that in the presence of tip–sample forces, the settling time can potentially be much longer. Such is the case when operating close to a bifurcation point between stable and unstable amplitude branches [21,31].

The feedback control plays an important role in determining the detection bandwidth in dAFM. As we have discussed, the auxiliary regulators in FM and DAM essentially have complete control over the oscillations of the probe, including the transient settling time. In [53], it was determined experimentally that FM and DAM can achieve similar detection bandwidths in vacuum. We remark that self-excited FM has a detection bandwidth of ω_0_ [8], but only when operating in the linear regime (a* << *λ*), which is rarely the case in high-resolution imaging. In a nonlinear regime, the frequency shift is coupled to the amplitude response [12], and the detection bandwidth is limited accordingly. Thus, the amplitude regulator in self-excited FM determines the overall detection bandwidth for high-resolution imaging when *B*_osc_ is small. On the other hand, the measurement bandwidth in AM is limited roughly by the oscillator bandwidth *B*_osc_.

Table 1 lists *B*_osc_ for high-resolution images in vacuum and liquid. The two AM references in vacuum achieve a relatively high bandwidth in vacuum by using nonstandard probes. Kawai and Kawakatsu [52] exploited a higher eigenmode of a silicon cantilever that had an unperturbed resonance frequency of 1.8 MHz. Erlandsson et al. [51] used a tungsten wire with an unperturbed quality factor of just 550 in vacuum. The references for FM in vacuum in Table 1 rely on feedback control to improve the detection bandwidth by about one order of magnitude over standard probes, yet still fall far short of our 1 kHz estimation. On the other hand, the low *Q*’s in liquids ensure that the bandwidth requirement is met without including the auxiliary feedback regulators (again, see Table 1). Consequently, AM is more successful at high-resolution imaging in liquids than in vacuum.

### Disturbance mitigation

A critical function of the feedback regulation in dAFM is to sustain the probe oscillations in the presence of unknown tip–sample forces, i.e., to mitigate disturbances from the tip–sample forces. In vacuum, the auxiliary regulators used in FM and DAM are essential for sustaining the oscillations in the presence of large noncontact tip–sample forces. Giessibl et al. [10] postulate that *e*_ts_ < 1 is required to maintain stable oscillations. However, the data in Figure 1 show that the auxiliary regulators are capable of maintaining stable oscillations when the magnitudes of *e*_ts_ and *v*_ts_ are much larger than unity. On the other hand, the approach taken in AM is simply to limit the magnitudes of *e*_ts_ and *v*_ts_, in order to keep the amplitude from being attenuated. For example, choosing *a*_sp_/*a*_0_ = 1/2 in AM requires that the magnitudes of *e*_ts_ and *v*_ts_ do not exceed unity according to Equation 6. This limited approach to feedback regulation in AM can be problematic in vacuum and air where noncontact forces are large, but it is generally sufficient for imaging in liquids.

### Stability

A final issue surrounding the high-resolution imaging in dAFM is stability. We have already discussed the importance of the auxiliary regulators in eliminating bistable oscillation states and maintaining stable oscillations in the presence of tip–sample forces. We turn now to the issue of global stability of the separation regulator. We first consider a stability issue that is inherent in FM, commonly referred to as “tip crash” [54]. To simplify matters, we require that *z* is manipulated slowly by the separation regulator such that the auxiliary feedback regulators maintain the probe oscillations in a quasi-steady state. Setting *K*_5_ = 0 and requiring *K*_6_ to be small in Equation 9, the dynamics of *z* in FM are approximated by

[20]
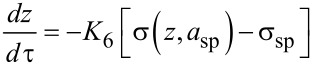


where σ_sp_ = −*v*_ts_(*z*,*a*_sp_).

**Figure 3:**
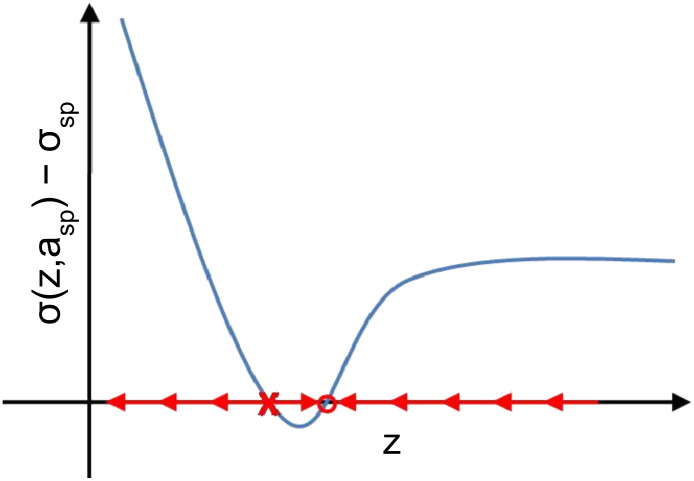
Stability of the *z* control in FM in vacuum. Arrows indicate the direction of the motion of *z* when placed under feedback control according to *dz*/*d*τ = −*K*_6_[σ(*z*,*a*_sp_) − σ_sp_], where *K*_6_ > 0.

The schematic in Figure 3 shows the typical behavior of σ(*z*,*a*_sp_) in vacuum or air where attractive forces are significant. The arrows indicate the direction in which *z* is instructed to move by the feedback regulator according to Equation 20. The equilibrium points z* are the zero crossings of σ(*z*,*a*_sp_) − σ_sp_. It is shown in Figure 3 that equilibrium in the attractive regime is locally stable but lacks global stability. A perturbation in the tip–sample forces can cause the separation regulator to approach the sample indefinitely. The closer σ_sp_ is to the onset of repulsive forces, the more likely is the onset of this instability. On the other hand, separation regulator in DAM is designed to maintain *e*_ts_. When the amplitude is constant, *e*_ts_ typically increases monotonically with respect to *z* (see Figure 1); a point that was originally made in [55]. Consequently, DAM allows the oscillating probe tip to pass through the boundary between attractive and repulsive forces without necessarily resulting in a loss of stability.

## Results and Discussion

In the previous sections, we investigated the role of the feedback control in high-resolution imaging. It is important to note that the task of regulating the oscillations of the probe under imaging conditions is greatly simplified in liquids due to the small long-range forces and low quality factors. Consequently, imaging modes with limited feedback control, such as AM, can be successful at high-resolution imaging in liquids [5]. In this section we present high-resolution images of mica in liquids with FM, DAM and AM acquired with the same probe and under similar operating conditions.

**Figure 4:**
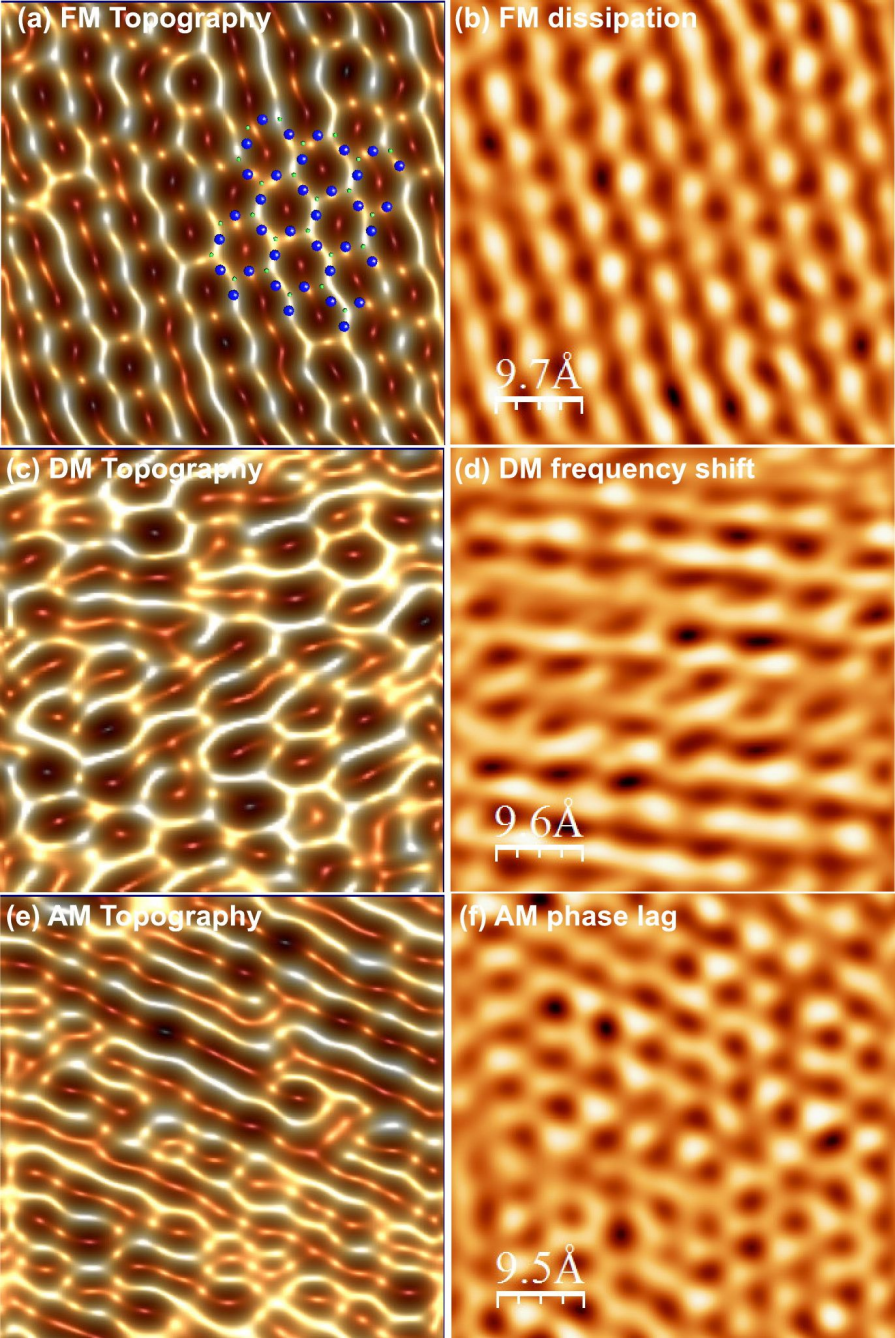
High-resolution images of mica in water taken with FM, DAM and AM. FM (a) topography and (b) dissipation. DAM (c) topography and (d) frequency-shift images. AM (e) topography and (f) phase-lag image. The variation in height in FM, DAM and AM topography images is 20, 80 and 40 pm, respectively, while the root-mean-squared surface roughness is 4, 13, and 6 pm, respectively. See Methods for additional specifications.

Figure 4 shows the topography and compositional images of freshly cleaved muscovite mica in water. Superimposed on the FM topography image is the theoretical structure of freshly cleaved muscovite mica, which exposes a plane of oxygen atoms (blue), which is slightly offset from a plane of silicon atoms (green) [2]. The FM dissipation image reveals a strong correlation with the FM topography image revealing dissipation on the atomic scale. The atomic-scale features in the FM dissipation image entertain the possibility of atomic-resolution imaging with DAM through regulation based on the tip–sample dissipation. This is indeed shown to be the case in Figure 4c. Similarly, atomic resolution is demonstrated in AM in Figure 4e. While topography images in AM reflect a combination of the dissipation and virial, AM is more sensitive to the dissipation at high amplitude set-points, in which case AM resembles DAM.

The primary difference between the topography images in FM, DAM and AM in liquids is the treatment of tip–sample forces by the separation regulator. Of the three imaging modes, it appears that FM most faithfully reproduces the expected theoretical structure of freshly cleaved mica. From the data presented, it appears that regulation of the conservative component of the interaction captured by the virial is more favorable for atomic-resolution imaging. Furthermore, from Figure 1, we note that the magnitude of the virial is often larger than the dissipation for stiff inorganic samples such as mica. However, we stress that it was possible to obtain high-resolution images of mica in water in each of the three imaging modes under similar operating conditions.

## Methods

The approach curves in Figure 1 were obtained by using the auxiliary regulators described in Equation 8, which is the typical precursory experiment to imaging with FM. The experimental data consist of *F* and σ versus *z*. Reconstruction of *e*_ts_ and *v*_ts_ versus *z* is achieved by substituting a* = *a*_0_ = *a*_sp_ and 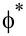 = π/2 into Equation 6 to yield

[21]
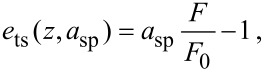


[22]
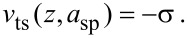


Measurements were made on a silicon substrate with 1 nm native silicon oxide in vacuum with *k* = 27 N/m, *Q*_0_ = 28,000 and *a*_sp_ = 8.5 nm and in ambient air with *k* = 36 N/m, *Q*_0_ = 620 and *a*_sp_ = 8.6 nm. Data were acquired on glass in deionized water with *k* = 0.6 N/m, *Q*_0_ = 1.6 and *a*_sp_ = 1.5 nm.

The high-resolution images of freshly cleaved mica in deionized water in Figure 4 were acquired with a Nanosenors™ PPP-NCH probe (*k* = 40 N/m, *Q*_0_ = 11). Images were obtained in FM with *a*_sp_ = 0.7 nm, σ_sp_ = 0.01, in AM with *a*_0_ = 0.6, *a*_sp_ = 0.86*a*_0_, and in DAM with *a*_sp_ = 0.4 and *F*_sp_ = 1.3*F*_0_. A wavelet filter with a scale of 0.13 nm was applied to each image by using the WSxM software [56]. The scan rate in the fast scan direction of the image raster is 440 nm/s, which was necessary to compensate for thermal drifts. For this scan rate, we calculate the required measurement bandwidth to be about 1 kHz for high-resolution imaging. All data were acquired with Nanotec Electrónica microscopes (Nanotec Eletronica S.L., Madrid, Spain) by using the WSxM software.

## Conclusion

Through analysis and experiment, we have studied the performance metrics for high-resolution imaging in liquids with different dAFM imaging modes. In general, we find that while the quality factors of probes in liquids are typically low, the force sensitivity can be preserved by using soft probes with small amplitudes. Remarkably, it is possible for a probe in liquid to have a force sensitivity on par with the qPlus sensor in vacuum. Moreover, we find that the reduction in both attractive forces and quality factors that occurs in liquids decreases the importance of feedback control in obtaining stable, high-resolution images in liquids. Thus, the considerable advantages of FM over AM in obtaining high-resolution images in vacuum are not reproduced in liquids. These findings are supported by high-resolution images of mica obtained with FM, AM and DAM in liquid under similar operating conditions. From the data, it does appear that FM still has some advantage over AM and DAM in atomic-resolution imaging. On the other hand, DAM offers robust stability for a range of environments and applications [53].
